# Using genotyping‐by‐sequencing to predict gender in animals

**DOI:** 10.1111/age.12782

**Published:** 2019-04-07

**Authors:** T. P. Bilton, A. J. Chappell, S. M. Clarke, R. Brauning, K. G. Dodds, J. C. McEwan, S. J. Rowe

**Affiliations:** ^1^ Invermay Agricultural Centre AgResearch Private Bag 50034 Mosgiel 9053 New Zealand

**Keywords:** allosomal SNPs, deer, low depth, sex assignment, XX/XY system

## Abstract

Gender assignment errors are common in some animal species and lead to inaccuracies in downstream analyses. Procedures for detecting gender misassignment are available for array‐based SNP data but are still being developed for genotyping‐by‐sequencing (GBS) data. In this study, we describe a method for using GBS data to predict gender using X and Y chromosomal SNPs. From a set of 1286 X chromosomal and 23 Y chromosomal deer (*Cervus* sp.) SNPs discovered from GBS sequence reads, a prediction model was built using a training dataset of 422 Red deer and validated using a test dataset of 868 Red deer and Wapiti deer. Prediction was based on the proportion of heterozygous genotypes on the X chromosome and the proportion of non‐missing genotypes on the Y chromosome observed in each individual. The concordance between recorded gender and predicted gender was 98.6% in the training dataset and 99.3% in the test dataset. The model identified five individuals across both datasets with incorrect recorded gender and was unable to predict gender for another five individuals. Overall, our method predicted gender with a high degree of accuracy and could be used for quality control in gender assignment datasets or for assigning gender when unrecorded, provided a suitable reference genome is available.

Gender misassignment, due to misidentification on examination, data input errors or genotyping errors, occurs in animal species and can hinder genetic gain in breeding programmes (Zhang *et al*. 2016). Some software packages, such as plink (Purcell *et al*. 2007) and pygenclean (Lemieux Perreault *et al*. 2013), contain quality control procedures to detect gender misassignment. These procedures generally use heterozygosity on the X chromosome to distinguish gender, because males, with only one X chromosome, should be hemizygous, whereas females can be heterozygous at some loci as they have two X chromosomes. One exception is the pseudo‐autosomal region (PAR; Johnston *et al*. 2017), a region of homology between the X and Y chromosomes where males can be heterozygous. Data in the PAR are usually discarded in SNP‐based gender prediction. There are limitations to using the X chromosome as the sole means of predicting gender, as sex anomalies can lead to incorrect assignment. Including Y chromosome information in the prediction may assist in correct assignment (Laurie *et al*. 2010; Turner *et al*. 2011; Zhang *et al*. 2016). Various gender tests have been developed for array‐based SNP calling platforms and typically focus on detecting sample handling errors in human genetic data (Anderson *et al*. 2010; Laurie *et al*. 2010; Turner *et al*. 2011). The high cost of SNP arrays prohibits their widespread use in many animal species, for which a cost‐efficient alternative is genotyping‐by‐sequencing (GBS). In this study, we developed a simple method for building a gender prediction model using X and Y chromosomal SNPs obtained from GBS data, using deer (*Cervus* sp.) as an example.

A set of allosomal (sex‐linked) SNPs were identified from GBS reads using the following procedure. A set of tag pairs, a collection of sequence reads that differ by a single base pair in the genomic part of a read, were identified using uneak (Lu *et al*. 2013) from GBS sequence reads in a large and diverse catalogue of deer samples. These tag pairs were aligned to a Red deer reference genome (Bana *et al*. 2018) using bowtie2 version 2.1.0 (Langmead & Salzberg 2012), with the parameter setting –very‐sensitive and custom Python scripts. SNPs located on tag pairs that aligned to the sex chromosomes were identified as allosomal; 1286 X chromosomal and 23 Y chromosomal SNPs were identified.

A training dataset of 422 deer, consisting predominantly of Red deer (*Cervus elaphus*) with some Red‐by‐Wapiti (*Cervus canadensis*) crosses, was used to build a prediction model, and a test dataset, consisting of 619 Red deer and 249 Wapiti, was used to validate the model, in which gender was recorded for all individuals. The animals were managed in accordance with the provisions of the New Zealand Animal Welfare Act 1999 and the Codes of Welfare developed under sections 68–79 of the Act. Tissue samples were collected using ear punches, and DNA was extracted according to Clarke *et al*. (2014). The GBS libraries were prepared using the restriction enzyme PstI following the method described by Elshire *et al*. (2011) with variations as outlined by Dodds *et al*. (2015). The individuals were sequenced at AgResearch, Invermay, Animal Genomics Laboratory on an Illumina HiSeq 2500 v4 chemistry yielding 100‐bp single end reads.

Using the training dataset, the identified allosomal SNPs were filtered to remove erroneous SNPs as follows. The X chromosomal SNPs were discarded if they were located in the PAR, if at least 10% of males showed heterozygosity or if at least 10% of females had missing genotypes. Using the data shown in Fig. S1, the PAR was inferred to commence at 170 Mb and extend to the telomere. For the Y chromosome, SNPs were discarded if at least 5% of females had non‐missing genotype calls or if at least 50% of males had heterozygous genotypes. These numbers were liberal to preserve more SNPs on the Y chromosome. Lastly, SNPs with a minor allele frequency less than 0.015 were removed as recommended by Zhang *et al*. (2016).

The samples for the test and training datasets with the filtered allosomal SNPs were run through kgd (https://github.com/AgResearch/KGD) using default settings, except that samples with a mean depth below 0.3 were discarded. Summary statistics of the filtered data are given in Table 1. For each individual, the proportion of Y chromosomal SNPs with non‐missing genotypes, $P_{Y_{i}}$, and the heterozygosity of the X chromosomal SNPs, *H*_*i*_, were computed. Heterozygosity was adjusted for read depth, using ideas from Dodds *et al*. (2015), to account for under‐called heterozygotes and was computed as $H_{i}=n_{i}/\sum_{j=1}^{M_{x}}\left(1-2K_{\mathrm{ij}}\right)$, where *n*_*i*_ is the number of heterozygous genotypes for individual *i*, $K_{\mathrm{ij}}=1/2^{d_{\mathrm{ij}}}$ and *d*_*ij*_ is the read depth at SNP *j* in individual *i* for *j* = 1, … , *M*_*x*_. Plots of $M_{y}P_{Y_{i}}$ against *H*_*i*_, which we refer to as gender plots, were produced, where *M*_*y*_ is the number of (filtered) Y chromosomal SNPs. The R code and the data used in this analysis are available at https://github.com/AgResearch/GBS_Gender_Predict.

**Table 1:** Summary statistics for the training and test datasets after being run through the kgd software

Dataset	Breed	# deer	# X SNPs	# Y SNPs	Recorded gender		Mean sample depth
					Females	Males	
Training	Red	422	1026	17	160	261	2.89
Test	Red	619	1010	16	305	314	1.52
Test	Wapiti	249			131	118	

The gender plot for the training dataset is given in Fig. 1a. In general, males were located in the upper left‐hand area of the plot and females along and slightly above the *x*‐axis. A prediction model was built using the empirical results from the training set such that an individual was classified male if $P_{Y_{i}}>20H_{i}^{2}+0.2$ or female if $P_{Y_{i}}<0.1+H_{i}$, otherwise gender was unassigned. Under this model, 98.6% of the predicted gender matched the recorded gender. Four recorded females were classified as male and one recorded male classified as female. The gender of these individuals was identified as phenotypic misassignments on re‐examination, with the prediction model correctly classifying each individual. The gender plot for the test dataset (Fig. 1b) gave results similar to those for the training dataset. The predicted gender of the Wapiti matched their recorded gender in all cases, except gender was not assigned for two recorded males. For the Red deer, two recorded males were not assigned gender, one recorded male was predicted female and one recorded female was predicted male. Upon re‐examination the latter two samples were identified as male. In total, 99.3% of the GBS‐predicted gender matched the recorded gender for the test dataset. For all the deer with consistent recorded and predicted gender, no erroneous recorded gender assignments, which become obvious as the animals mature, have been discovered by the breeders.

**Figure 1:**
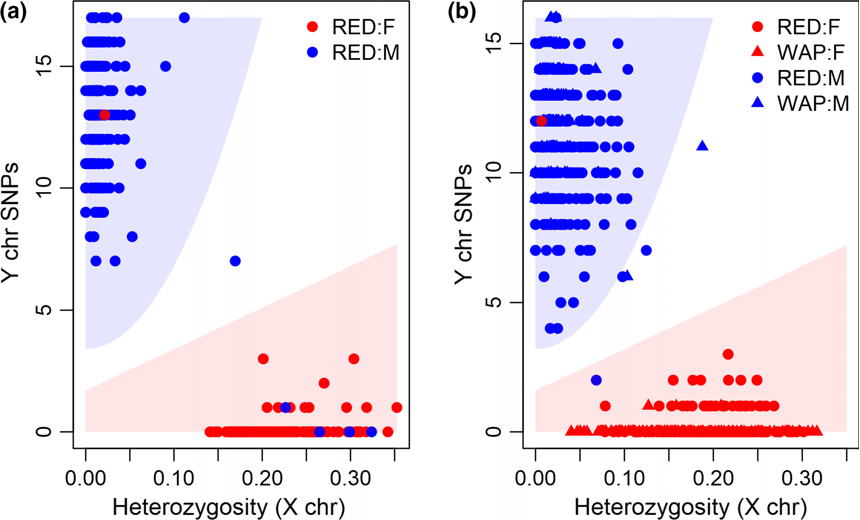
Gender plot for the (a) training dataset and (b) test dataset. Red symbols represent recorded females, blue symbols represent recorded males, circles represent Red deer and triangles represent Wapiti deer. Animals were predicted male if located in the blue region [$M_{y}\left(P_{Y_{i}}>20H_{i}^{2}+0.2\right)$], were predicted female if located in the red region [$M_{y}\left(P_{Y_{i}}<\;0.1+H_{i}\right)$] or otherwise were unassigned gender. Interactive plots of (a) and (b) can be downloaded at https://github.com/AgResearch/GBS_Gender_Predict/tree/master/Supplementary/.

Overall, the accuracy of our method was 99.9% with a specificity (true male predicted male) and sensitivity (true female predicted female) of 99.8% and 100% respectively. These measures are similar to those obtained using SNP chip arrays (Zhang *et al*. 2016) but higher than traditional sexing assays (such as PCR‐based approaches or capillary electrophoresis; Wright *et al*. 2012; Ristanic *et al*. 2018) and other sequencing‐based approaches (Stovall *et al*. 2018). However, unlike SNP chip and traditional sexing assays, the GBS‐based method is applicable across different species, does not require a costly development phase and is a by‐product of having GBS SNP data with their chromosomal positions.

In this work, we developed a method for discovering SNPs located on sex chromosomes in GBS data and using them to predict gender. Results show that the predictions were highly accurate, even across two diverse breeds of deer, and lead to the detection of animals with wrongly recorded gender. Furthermore, the results demonstrate that using both X and Y chromosomes improves accuracy in gender prediction with GBS data compared to using solely the X chromosome information. This is due to the presence of errors (e.g. alignment or sequencing errors) that result in males having non‐zero heterozygosity estimates for X chromosomal SNPs and reads to be called on the Y chromosome for females. Nevertheless, by combining information from the X and Y chromosomes, sufficient differentiation between males and females was obtained in order to predict gender accurately.

One advantage of our method is that it accounts for errors from heterozygous genotypes being called as homozygous in the calculation of heterozygosity, which allows gender prediction on samples with low sample depth. Nevertheless, we discarded individuals with a sample depth below 0.3 because extremely low sample depth can lead to very few or no reads being observed for Y chromosomal SNPs. This would result in males having unassigned gender or even being classified female, as only a few Y chromosomal SNPs were discovered. This problem could be mitigated by using additional sequence reads without SNPs that align to the Y chromosome. However, this raw sequence information is often not available to the end user.

For some individuals, points on the gender plot may fall outside the male and female regions. This may happen if the animal's sample depth is low, as errors can bias heterozygosity estimates for males. In our analysis, all but one of the individuals with unassigned gender were males with a sample depth around 0.6 or lower. Alternatively, these points may represent sex chromosome aneuploidies, such as males with XXY (Laurie *et al*. 2010; Turner *et al*. 2011). A female with karyotype XO aneuploidy (Berry *et al*. 2017) is likely to be classified as female under our model, as $P_{Y_{i}}$, and *H*_*i*_ would be expected to equal zero. Another possibility is that sample contamination may lead to unexpected values of $P_{Y_{i}}$ and *H*_*i*_, although this would depend on the type and level of contamination present. Consequently, our method could be utilised to detect aneuploidies or contaminated samples. Without any known abnormal animals or samples available in our collection, this could not be tested.

In conclusion, we have developed a method using GBS data that predicts gender in animals with a high degree of accuracy. Although developed in deer, our method can be readily applied to other animal species with an XX/XY sex determination system, provided that a suitable reference genome with X and Y chromosomes is available.

## Supporting information


**Figure S1** Proportion of heterozygous genotypes for males in the training dataset for X chromosomal SNPs relative to their position on the X chromosome.

## References

[age12782-bib-0001] Anderson C.A. , Pettersson F.H. , Clarke G.M. , Cardon L.R. , Morris A.P. & Zondervan K.T. (2010) Data quality control in genetic case‐control association studies. Nature Protocols 5, 1564–73. 21085122 10.1038/nprot.2010.116 PMC3025522

[age12782-bib-0002] Bana N.Á. , Nyiri A. , Nagy J. *et al* (2018) The red deer *Cervus elaphus* genome CerEla1.0: sequencing, annotating, genes, and chromosomes. Molecular Genetics and Genomics 293, 665–84. 29294181 10.1007/s00438-017-1412-3

[age12782-bib-0003] Berry D.P. , Wolfe A. , O'Donovan J. , Byrne N. , Sayers R.G. , Dodds K.G. , McEwan J.C. , O'Connor R.E. , McClure M. & Purfield D.C. (2017) Characterization of an X‐chromosomal non‐mosaic monosomy (59, X0) dairy heifer detected using routinely available single nucleotide polymorphism genotype data. Journal of Animal Science 95, 1042–9. 28380529 10.2527/jas.2016.1279

[age12782-bib-0004] Clarke S.M. , Henry H.M. , Dodds K.G. , Jowett T.W. , Manley T.R. , Anderson R.M. & McEwan J.C. (2014) A high throughput single nucleotide polymorphism multiplex assay for parentage assignment in New Zealand sheep. PLoS ONE 9, e93392. 24740141 10.1371/journal.pone.0093392 PMC3989167

[age12782-bib-0005] Dodds K.G. , McEwan J.C. , Brauning R. , Anderson R.M. , Stijn T.C. , Kristjánsson T. & Clarke S.M. (2015) Construction of relatedness matrices using genotyping‐by‐sequencing data. BMC Genomics 16, 1047. 26654230 10.1186/s12864-015-2252-3 PMC4675043

[age12782-bib-0006] Elshire R.J. , Glaubitz J.C. , Sun Q. , Poland J.A. , Kawamoto K. , Buckler E.S. & Mitchell S.E. (2011) A robust, simple genotyping‐by‐sequencing (GBS) approach for high diversity species. PLoS ONE 6, e19379. 21573248 10.1371/journal.pone.0019379 PMC3087801

[age12782-bib-0007] Johnston S.E. , Huisman J. , Ellis P.A. & Pemberton J.M. (2017) A high‐density linkage map reveals sexual dimorphism in recombination landscapes in red deer (*Cervus elaphus*). G3: Genes, Genomes, Genetics 7, 2859–70. 28667018 10.1534/g3.117.044198 PMC5555489

[age12782-bib-0008] Langmead B. & Salzberg S.L. (2012) Fast gapped‐read alignment with bowtie 2. Nature Methods 9, 357. 22388286 10.1038/nmeth.1923 PMC3322381

[age12782-bib-0009] Laurie C.C. , Doheny K.F. , Mirel D.B. *et al* (2010) Quality control and quality assurance in genotypic data for genome‐wide association studies. Genetic Epidemiology 34, 591–602. 20718045 10.1002/gepi.20516 PMC3061487

[age12782-bib-0010] Lemieux Perreault L.P. , Provost S. , Legault M.A. , Barhdadi A. & Dubé M.P. (2013) pygenclean: efficient tool for genetic data clean up before association testing. Bioinformatics 29, 1704–5. 23652425 10.1093/bioinformatics/btt261 PMC3694635

[age12782-bib-0011] Lu F. , Lipka A.E. , Glaubitz J. , Elshire R. , Cherney J.H. , Casler M.D. , Buckler E.S. & Costich D.E. (2013) Switchgrass genomic diversity, ploidy, and evolution: novel insights from a network‐based SNP discovery protocol. PLoS Genetics 9, e1003215. 23349638 10.1371/journal.pgen.1003215 PMC3547862

[age12782-bib-0012] Purcell S. , Neale B. , Todd‐Brown K. *et al* (2007) plink: a tool set for whole‐genome association and population‐based linkage analyses. American Journal of Human Genetics 81, 559–75. 17701901 10.1086/519795 PMC1950838

[age12782-bib-0013] Ristanic M. , Stanisic L. , Maletic M. , Glavinic U. , Draskovic V. , Aleksic N. & Stanimirovic Z. (2018) Bovine foetal sex determination—different DNA extraction and amplification approaches for efficient livestock production. Reproduction in Domestic Animals 53, 947–54. 29740884 10.1111/rda.13193

[age12782-bib-0014] Stovall W.R. , Taylor H.R. , Black M. , Grosser S. , Rutherford K. & Gemmell N.J. (2018) Genetic sex assignment in wild populations using genotyping‐by‐sequencing data: a statistical threshold approach. Molecular Ecology Resources 18, 179–90. 29443461 10.1111/1755-0998.12767

[age12782-bib-0015] Turner S. , Armstrong L.L. , Bradford Y. *et al* (2011) Quality control procedures for genome‐wide association studies. Current Protocols in Human Genetics 68, 1.19.1–8. 10.1002/0471142905.hg0119s68 PMC3066182 21234875

[age12782-bib-0016] Wright C.F. , Wei Y. , Higgins J.P. & Sagoo G.S. (2012) Non‐invasive prenatal diagnostic test accuracy for fetal sex using cell‐free DNA a review and meta‐analysis. BMC Research Notes 5, 476. 22937795 10.1186/1756-0500-5-476 PMC3444439

[age12782-bib-0017] Zhang I.L. , Couldrey C. & Sherlock R.G. (2016) Using genomic information to predict sex in dairy cattle. Proceedings of the New Zealand Society of Animal Production 76, 26–30.

