# Supplementary material for: Using genotyping‐by‐sequencing to predict gender in animals
Source: Anim Genet. 2019 Apr 7;50(3):307–10. doi: 10.1111/age.12782 (PMC6593676; doi:10.1111/age.12782)

Figure S1 Proportion of heterozygous genotypes for males in the training dataset for X chromosomal SNPs relative to their position on the X chromosome. Subplot (a) shows all the SNPs before filtering and (b) the SNPs after filtering. The solid red vertical line in subplot (a) represents the inferred start of the PAR region at 170 Mb and the dashed red horizontal line represents the filter of removing X chromosomal SNPs where at least 10% of the males are heterozygous.

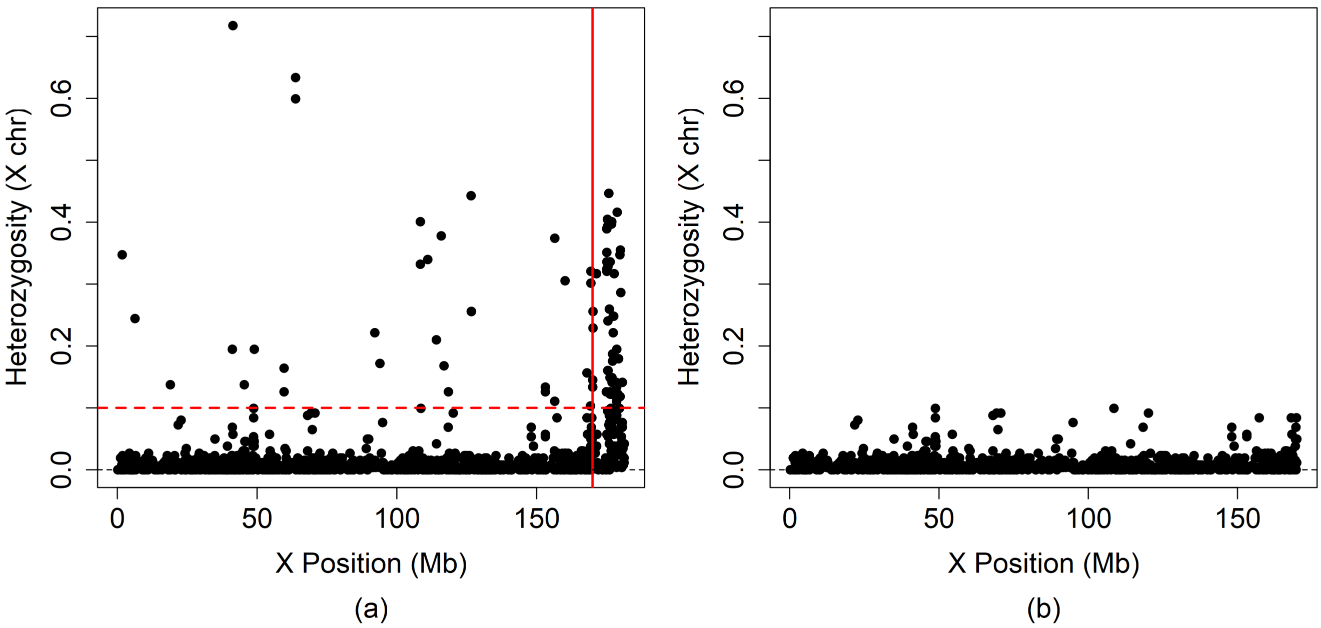

Supplement: Supplementary file 1 — Figure S1 Proportion of heterozygous genotypes for males in the training dataset for X chromosomal SNPs relative to their position on the X chromosome. [file AGE-50-307-s001.pdf]
